# Mucocutaneous Hyperpigmentation as the Diagnostic Clue to Autoimmune Polyglandular Syndrome Type 2: A Case Report

**DOI:** 10.7759/cureus.114347

**Published:** 2026-08-11

**Authors:** Pedro Reboredo, Tiago Resende, Paula Nogueira, Cristina Sousa, Ramiro Lopes

**Affiliations:** 1 Internal Medicine, Unidade Local de Saúde do Algarve, Hospital de Faro, Faro, PRT; 2 Nephrology, Unidade Local de Saúde do Algarve, Hospital de Faro, Faro, PRT

**Keywords:** addison disease, adrenal crisis, autoimmune polyglandular syndrome type 2, autoimmune thyroiditis, mucocutaneous hyperpigmentation, physical examination, primary adrenal insufficiency

## Abstract

Autoimmune polyglandular syndrome type 2 (APS-2) is a rare autoimmune disorder characterized by the coexistence of primary adrenal insufficiency with autoimmune thyroid disease and/or type 1 diabetes mellitus. Because its initial manifestations are often nonspecific, diagnosis may be substantially delayed until advanced disease or adrenal crisis develops. We report the case of a 28-year-old Portuguese woman living in California who presented with a nine-month history of progressive fatigue, unintentional weight loss, nausea, vomiting, abdominal pain, and a previous syncopal episode. Recognition of diffuse mucocutaneous hyperpigmentation involving the palmar creases and tongue prompted targeted endocrine investigation. Laboratory evaluation confirmed autoimmune primary adrenal insufficiency and autoimmune hypothyroidism, establishing the diagnosis of APS-2. Treatment with intravenous hydrocortisone and fluid resuscitation was followed by levothyroxine and fludrocortisone replacement, resulting in rapid clinical improvement, the normalization of biochemical abnormalities, and complete recovery. This case highlights the diagnostic value of meticulous physical examination in patients presenting with otherwise unexplained constitutional symptoms. Recognition of these characteristic physical findings may facilitate the early diagnosis of autoimmune primary adrenal insufficiency, enabling appropriate hormone replacement before progression to adrenal crisis.

## Introduction

Autoimmune polyglandular syndrome type 2 (APS-2) is the most common autoimmune polyendocrine syndrome in adults and is characterized by the coexistence of primary adrenal insufficiency with autoimmune thyroid disease and/or type 1 diabetes mellitus [1,2]. Nevertheless, it remains rare, predominantly affects women, and typically presents during the third or fourth decade of life [1,3]. Primary adrenal insufficiency is the defining and potentially life-threatening component of the syndrome. However, its diagnosis remains challenging because the initial presentation is often insidious and dominated by nonspecific constitutional and gastrointestinal symptoms, frequently resulting in delayed recognition [2-5].

Diffuse mucocutaneous hyperpigmentation is a characteristic feature of primary adrenal insufficiency and may represent one of the earliest clinical manifestations, although it is easily overlooked or attributed to more common causes, particularly in individuals with significant sun exposure [2,4,6]. Prompt recognition is essential because early diagnosis allows appropriate glucocorticoid replacement before thyroid hormone therapy, preventing adrenal crisis and improving clinical outcomes [3,4]. We report the case of a young woman in whom recognition of diffuse mucocutaneous hyperpigmentation during physical examination redirected the diagnostic workup toward autoimmune primary adrenal insufficiency after several months of unexplained constitutional symptoms, ultimately leading to the diagnosis of APS-2 and the timely initiation of hormone replacement therapy. This case highlights the enduring value of meticulous physical examination in identifying uncommon but potentially life-threatening endocrine disorders.

## Case presentation

A 28-year-old Portuguese woman with no relevant past medical history presented to the emergency department with a nine-month history of progressive constitutional symptoms, including fatigue, generalized weakness, nausea, vomiting, diffuse abdominal pain, palpitations, and an unintentional 11-kg weight loss, corresponding to approximately 21% of her usual body weight. Three months before presentation, she experienced a syncopal episode. During the weeks preceding admission, she developed marked exercise intolerance and dizziness. The patient had been living and working in California, USA, where the gradual skin hyperpigmentation went largely unnoticed because she initially attributed it to regular sun exposure.

**Figure 1:**
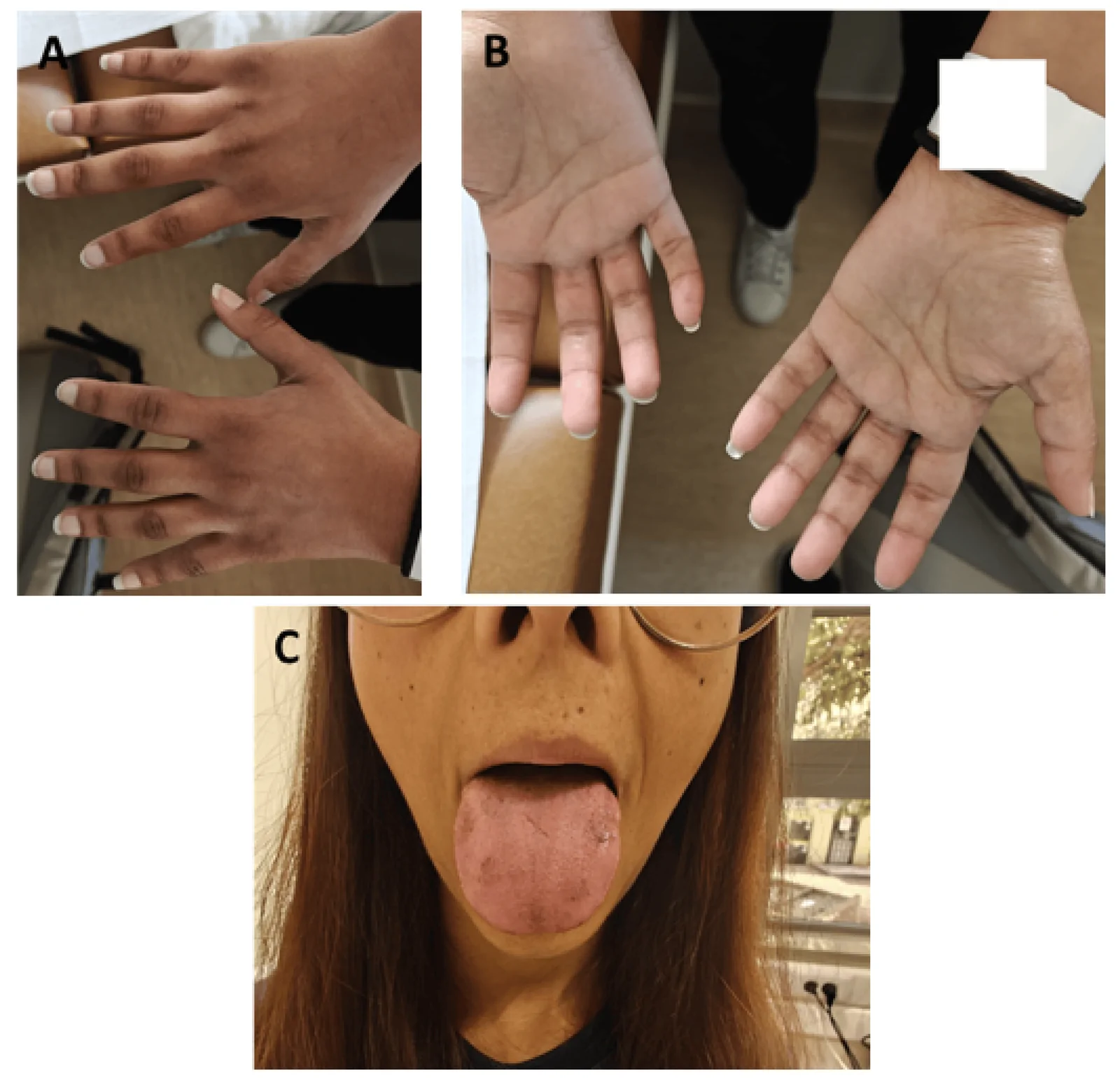
Characteristic mucocutaneous hyperpigmentation at presentation (A) Diffuse hyperpigmentation of the dorsal hands. (B) Hyperpigmentation accentuated over the palmar creases. (C) Patchy hyperpigmentation involving the dorsal surface of the tongue. These findings constituted an important clinical clue leading to the suspicion of primary adrenal insufficiency.

On admission, she was alert and fully oriented but appeared chronically ill. She was hypotensive (blood pressure 77/51 mmHg), with a heart rate of 88 beats/minute, a body temperature of 36°C, and an oxygen saturation of 98% while breathing ambient air. Physical examination revealed striking diffuse mucocutaneous hyperpigmentation involving both sun-exposed and non-sun-exposed areas, particularly the palmar creases, interdigital folds, axillae, popliteal fossae, and dorsal surface of the tongue (Figure 1). The remainder of the physical examination, including cardiopulmonary, abdominal, and neurological assessment, was unremarkable.

These physical findings, together with persistent hypotension and constitutional symptoms, prompted investigation for an underlying endocrine disorder. Initial laboratory evaluation demonstrated hyponatremia, mild hyperkalemia, and hypochloremia. Thyroid function tests revealed severe primary hypothyroidism with a thyroid-stimulating hormone (TSH) concentration of 200 μIU/mL and free thyroxine (fT4) of 0.34 ng/dL. Further endocrine evaluation showed markedly elevated adrenocorticotropic hormone (ACTH) levels (1,679 pg/mL), low serum cortisol concentrations, and strongly positive anti-thyroid peroxidase antibodies, confirming primary adrenal insufficiency associated with autoimmune thyroiditis. Testing for 21-hydroxylase antibodies was not available during the patient's diagnostic workup. The principal laboratory findings are summarized in Table 1.

**Table 1:** Initial laboratory findings on admission *Sample obtained before corticosteroid replacement. †Evening reference interval according to the local laboratory.

Laboratory parameter	Result	Reference range
Sodium	128 mmol/L	136-144 mmol/L
Potassium	5.2 mmol/L	3.3-5.1 mmol/L
Chloride	94 mmol/L	101-111 mmol/L
Blood urea nitrogen	28 mg/dL	7-18.7 mg/dL
Creatinine	1.1 mg/dL	0.6-1.1 mg/dL
Thyroid-stimulating hormone	200 μIU/mL	0.35-4.94 μIU/mL
Free T4	0.34 ng/dL	0.93-1.70 ng/dL
Basal adrenocorticotropic hormone	1,679 pg/mL	7.2-63.3 pg/mL
Serum cortisol*	1.7 μg/dL	2.5-12.5† μg/dL
Anti-thyroid peroxidase antibodies	>600 IU/mL	<34 IU/mL

The combination of diffuse cutaneous and mucosal hyperpigmentation, persistent hypotension, electrolyte abnormalities, and hormonal profile established the diagnosis of APS-2, comprising autoimmune primary adrenal insufficiency and autoimmune hypothyroidism.

Treatment was initiated with intravenous isotonic saline and hydrocortisone replacement. Levothyroxine was introduced only after adequate glucocorticoid replacement had been established, followed by fludrocortisone for mineralocorticoid replacement. The patient showed rapid clinical improvement, with normalization of blood pressure and serum sodium concentrations, resolution of gastrointestinal symptoms, and progressive recovery of functional capacity. She was subsequently transferred to her local referral hospital for continuation of inpatient care and discharged on hydrocortisone, fludrocortisone, and levothyroxine replacement therapy.

**Figure 2:**
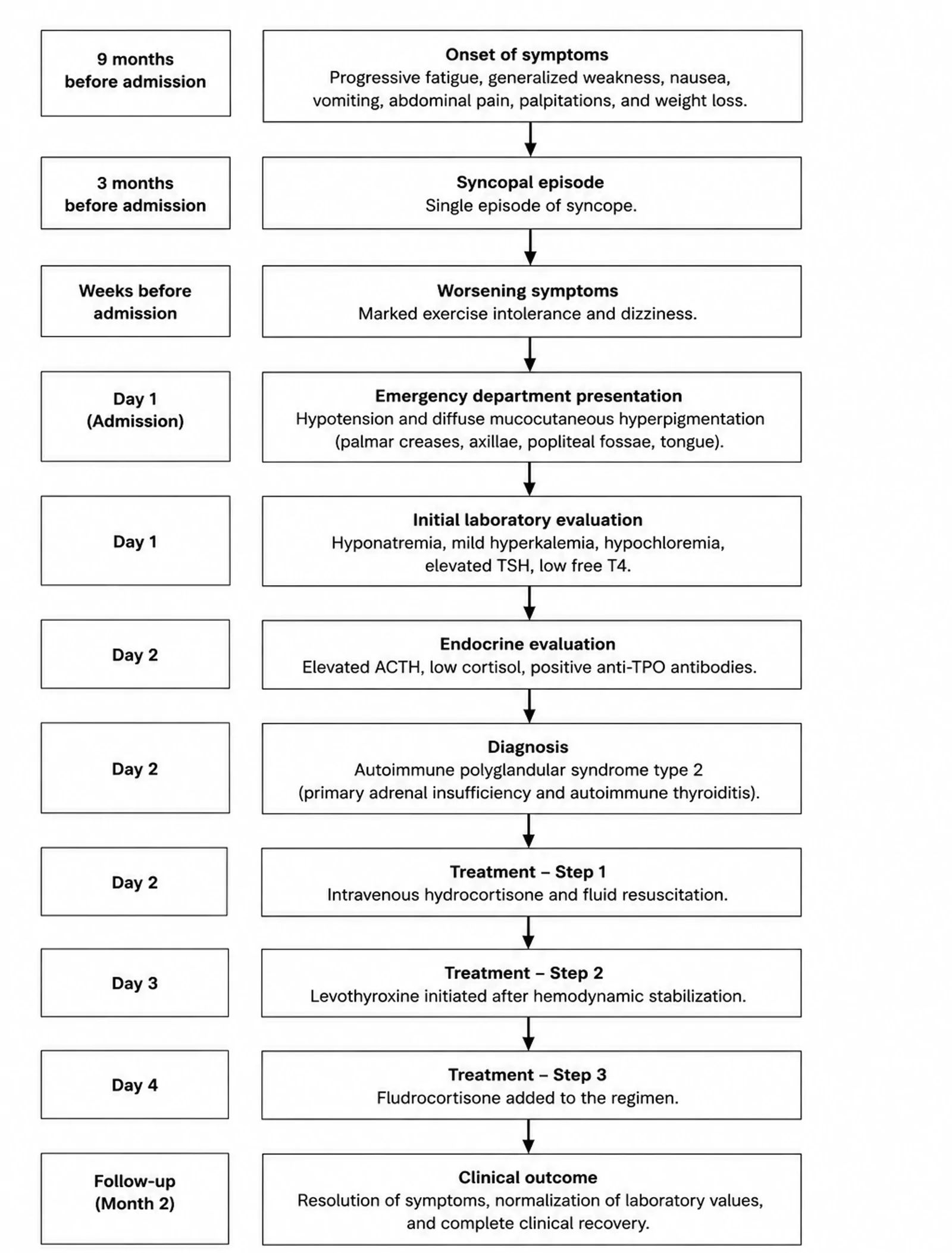
Timeline of symptom evolution, diagnostic workup, treatment, and clinical outcome TSH: thyroid-stimulating hormone; ACTH: adrenocorticotropic hormone; anti-TPO: anti-thyroid peroxidase Image created by the authors using Microsoft PowerPoint (Microsoft Corporation, Redmond, Washington, United States).

During the outpatient follow-up, she remained clinically stable with progressive normalization of thyroid function, recovery of her usual body weight, and return to regular physical activity without recurrence of symptoms. The chronological sequence of symptom onset, diagnostic workup, treatment, and clinical recovery is summarized in Figure 2.

## Discussion

The principal teaching point of this case is not the rarity of APS-2, but the diagnostic value of careful physical examination in a patient presenting with progressive yet nonspecific constitutional symptoms. Fatigue, weight loss, nausea, vomiting, and abdominal discomfort are common complaints encountered in Internal Medicine and frequently lead to extensive investigations before an endocrine disorder is considered. Consequently, the diagnosis of primary adrenal insufficiency is often delayed, sometimes until adrenal crisis develops [2-5]. Our patient's nine-month clinical course, including repeated medical assessments and a syncopal episode before the diagnosis was established, closely reflects this well-recognized diagnostic challenge.

Previous reports of APS-2 have consistently highlighted the diagnostic challenges posed by its nonspecific constitutional symptoms, with many patients undergoing multiple medical evaluations before the diagnosis is established [1,2,5,6]. Similar to these reports, our patient experienced several months of progressive symptoms before autoimmune primary adrenal insufficiency was recognized. However, this case particularly illustrates how meticulous physical examination and recognition of diffuse mucocutaneous hyperpigmentation redirected the diagnostic workup, allowing prompt endocrine evaluation and the timely initiation of hormone replacement before progression to adrenal crisis [2,4].

Recognition of diffuse hyperpigmentation involving the palmar creases, tongue, and other non-sun-exposed areas fundamentally changed the diagnostic approach by immediately raising suspicion of primary adrenal insufficiency. Hyperpigmentation is a characteristic manifestation of chronic ACTH excess and remains one of the most valuable bedside findings in patients with adrenal insufficiency [2,6,7]. In this case, however, progressive skin darkening had been attributed to regular sun exposure while living and working in California, illustrating how an important clinical sign may be overlooked when interpreted outside its clinical context. Diffuse mucocutaneous hyperpigmentation may also occur in conditions such as hereditary haemochromatosis, drug-induced pigmentation, Peutz-Jeghers syndrome, and ectopic ACTH syndrome. However, in our patient, the combination of persistent hypotension, characteristic electrolyte abnormalities, markedly elevated ACTH concentrations, low serum cortisol levels, and autoimmune thyroid disease strongly supported the diagnosis of autoimmune primary adrenal insufficiency. This observation emphasizes the importance of examining non-sun-exposed areas, particularly the palmar creases and oral mucosa, in patients presenting with otherwise unexplained constitutional symptoms.

The coexistence of autoimmune hypothyroidism and primary adrenal insufficiency established the diagnosis of APS-2 and had immediate therapeutic implications. Thyroid hormone replacement increases metabolic demand and accelerates cortisol clearance, potentially precipitating adrenal crisis in patients with untreated primary adrenal insufficiency. Current international guidelines therefore recommend initiating glucocorticoid replacement before levothyroxine whenever adrenal insufficiency is suspected or confirmed [4]. Our patient's management followed this approach, with hydrocortisone and fluid resuscitation preceding thyroid hormone replacement, resulting in rapid clinical stabilization and complete recovery.

Long-term follow-up remains essential because patients with APS-2 are at increased risk of developing additional autoimmune diseases throughout life, including type 1 diabetes mellitus, pernicious anemia, celiac disease, premature ovarian insufficiency, and vitiligo [1,3,8]. Accordingly, continued multidisciplinary surveillance and patient education regarding adrenal crisis prevention are fundamental components of long-term management.

This case demonstrates how meticulous physical examination can transform a constellation of apparently unrelated symptoms into a unifying diagnosis. Recognition of characteristic pigmentation prompted timely endocrine investigation and appropriate hormone replacement, preventing progression to adrenal crisis. Although APS-2 is uncommon, this report reinforces a broader principle applicable to everyday Internal Medicine: careful bedside examination remains indispensable for identifying potentially life-threatening diseases that may initially present with nonspecific clinical manifestations.

## Conclusions

APS-2 should be considered in patients presenting with unexplained constitutional symptoms associated with diffuse mucocutaneous hyperpigmentation, particularly when accompanied by hypotension or electrolyte abnormalities. This case demonstrates how careful physical examination can provide the crucial diagnostic clue, enabling the timely recognition of primary adrenal insufficiency and appropriate hormone replacement before progression to adrenal crisis. Beyond illustrating a rare autoimmune syndrome, this case reinforces the educational value of meticulous bedside examination in facilitating the early recognition of potentially life-threatening endocrine disorders.
